# Association Between Contrasting Water Regimes and Telomere Length Variation in Field-Grown Grapevines: An Integrated Physiological, Metabolomic and Molecular Approach

**DOI:** 10.3390/plants15131988

**Published:** 2026-06-26

**Authors:** Alessandra Iannuzzi, Ramona Pistucci, Arturo Erbaggio, Rossella Albrizio, Andrea Vitale, Filippo Accomando, Maurizio Buonanno, Antonio Dario Troise, Sabrina De Pascale, Antonello Bonfante

**Affiliations:** 1 Institute for the Animal Production System in the Mediterranean Environment (ISPAAM), National Research Council of Italy (CNR), 80055 Portici, Italy; ramonapistucci@cnr.it (R.P.); antoniodario.troise@cnr.it (A.D.T.); sabrina.depascale@cnr.it (S.D.P.); 2 Independent Researcher, 83100 Avellino, Italy; arturo.erbaggio@gmail.com; 3 Institute for Mediterranean Agricultural and Forest Systems (ISAFOM), National Research Council of Italy (CNR), 80055 Portici, Italy; rossella.albrizio@cnr.it (R.A.); andrea.vitale@cnr.it (A.V.); filippoaccomando@cnr.it (F.A.); maurizio.buonanno@cnr.it (M.B.); antonello.bonfante@cnr.it (A.B.)

**Keywords:** grapevine, telomere length, irrigation regimes, qPCR, metabolomics

## Abstract

Climate change is increasing the exposure of crops to drought stress, highlighting the need for integrative approaches to assess plant responses under field conditions. In this study, telomere length (TL) was evaluated in field-grown grapevine (*Vitis vinifera* L., cv. Aglianico) subjected to rainfed (RF) and controlled deficit irrigation (CDI) regimes. A qPCR-based protocol was applied together with physiological measurements, UAV-derived vegetation indices, and berry metabolomic profiling to investigate plant responses to different water regimes. Physiological and metabolomic analyses confirmed distinct responses between treatments, with rainfed vines showing more negative leaf water potential, lower stomatal conductance, and increased accumulation of stress-associated metabolites, including anthocyanins and abscisic acid. Linear mixed-effects modeling showed no significant difference in TL between treatments at the beginning of the experimental period (*p* = 0.198), whereas rainfed vines displayed significantly lower TL values than irrigated vines at the end of the growing season (*p* = 0.0009). TL decreased significantly over time in both treatments. The treatment × time interaction suggested a greater TL reduction in rainfed vines in the primary model (*p* = 0.064), and this effect was significant in a complete-pair sensitivity analysis (*p* = 0.036). These findings indicate an association between irrigation regime and telomere length variation under field conditions. The study provides preliminary evidence supporting the potential application of TL measurements for investigating plant responses to environmental stress in grapevine.

## 1. Introduction

Climate change is increasingly affecting agricultural systems worldwide, exposing crops to more frequent and prolonged drought periods and reducing water availability for irrigation [1]. In Mediterranean environments, where viticulture represents a major economic and cultural sector, grapevine (*Vitis vinifera* L.) cultivation is particularly vulnerable to fluctuations in water availability and increasing temperatures. Water deficit can strongly influence grapevine physiology, berry metabolism, and fruit composition, ultimately affecting grape and wine quality [2,3]. Under these conditions, the development of reliable approaches for monitoring plant responses to drought stress has become increasingly important for supporting sustainable vineyard management and improving crop adaptation to changing environmental conditions.

Several methods are currently used to assess grapevine water status and stress responses under field conditions. Physiological measurements such as leaf water potential (LWP) and stomatal conductance are among the most widely adopted indicators of plant water status and gas exchange regulation during drought stress. In parallel, remote sensing technologies based on unmanned aerial vehicles (UAVs) have become increasingly important in viticulture, allowing rapid and non-destructive evaluation of canopy vigor and spatial variability through multispectral vegetation indices [4]. In addition, metabolomic analyses provide detailed information on biochemical and metabolic responses associated with abiotic stress, including changes in secondary metabolism and phytohormone accumulation [5,6]. Together, these approaches provide complementary information on plant physiological and metabolic adjustments under water-limited conditions.

Despite their usefulness, these methods mainly describe short-term physiological responses or condition-specific metabolic changes and often require repeated measurements, specialized instrumentation, or complex data processing. Consequently, increasing interest has emerged in identifying molecular indicators potentially associated with environmental stress responses in plants. Among these, telomere biology has recently attracted attention in both animal and plant systems.

Telomeres are repetitive DNA sequences located at chromosome ends that preserve chromosome integrity and genome stability [7]. In plants, telomere dynamics are influenced by several biological processes, including cell division, developmental stage, aging, and environmental stress exposure. In particular, telomeric regions are considered highly sensitive to oxidative damage because of their guanine-rich sequences [8]. Previous studies in plants reported associations between telomere dynamics and developmental or environmental responses in species such as Arabidopsis thaliana, almond, and poplar [8,9,10]. In addition, natural variation in telomere length has been associated with adaptive and life-history traits in several plant species [11]. However, despite increasing interest in plant telomere biology, relatively few studies have explored telomere length (TL) variation under field conditions in crop plants exposed to contrasting environmental conditions, and evidence in perennial crops such as grapevine remains particularly limited.

qPCR-based methods are widely used to estimate TL because they allow rapid and relatively inexpensive analysis using small amounts of genomic DNA [12]. Nevertheless, the interpretation of TL variation in plants remains challenging, particularly under heterogeneous environmental conditions and across different cultivars or species [13]. Telomere dynamics can be influenced by multiple biological and technical factors, and the relationship between environmental stress and TL variation is still incompletely understood. Additional studies conducted under field conditions are therefore needed to better evaluate whether TL variation may be associated with plant responses to abiotic stress in agricultural systems.

Grapevine represents a particularly interesting model for investigating these relationships because irrigation management strongly affects vine physiology, berry metabolism, and fruit composition. Water deficit is known to induce physiological and biochemical responses associated with oxidative and metabolic stress, including changes in stomatal regulation, photosynthetic activity, secondary metabolism, and phytohormone accumulation [5,6]. Integrating molecular approaches with physiological and metabolomic analyses may therefore improve the understanding of plant responses to contrasting irrigation conditions in vineyard systems. Furthermore, recent studies highlighted the growing interest in molecular and cytogenetic approaches for investigating genome stability and stress-associated responses in biological systems [14].

In this study, we investigated the association between different water regimes and TL variation in field-grown grapevine (*Vitis vinifera* L., cv. Aglianico).

To achieve this aim, vines subjected to rainfed (RF) and controlled deficit irrigation (CDI) treatments were evaluated through an integrated approach combining physiological measurements, UAV-derived vegetation indices, berry metabolomic profiling, and qPCR-based TL analysis. Attention was given to evaluating whether TL variation was associated with drought-induced responses, aiming to understand if it could provide complementary information to conventional physiological and biochemical indicators of plant water status.

## 2. Results

### 2.1. Plant Responses

The two treatments, RF (rainfed) and CDI (controlled deficit irrigation), did not differ until 23 June, showing similar leaf water potential and stomatal conductance values. During the period from 1 April to 23 June, cumulative rainfall amounted to 128 mm, whereas reference evapotranspiration (ET_0_) reached 316 mm. Following the onset of irrigation, the two treatments began to diverge. Between 26 June and 16 September, the CDI treatment received a total of 506 mm of irrigation water, while cumulative reference evapotranspiration was 306 mm, and rainfall contributed an additional 104 mm.

As expected, water application led to statistically different vine water status between the two treatments (*p* < 0.05); the LWP mean value from 26 June to 16 September was −1.49 ± 0.16 MPa for RF treatment and −1.27 ± 0.25 MPa for CDI treatment. This behavior also impacted stomatal conductance, with mean values of 0.200 ± 0.07 mol m^−2^ s^−1^ for the RF treatment and 0.291 ± 0.09 mol m^−2^ s^−1^ for CDI.

**Figure 1:**
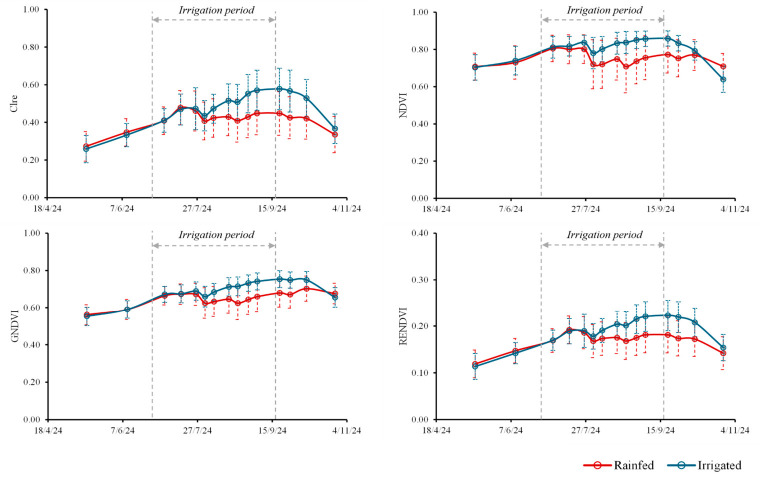
Trends of the multispectral indices Clre (Chlorophyll Index Red Edge), GNDVI (Green Normalized Difference Vegetation Index), NDVI (Normalized Difference Vegetation Index), and RENDVI (Red Edge Normalized Difference Vegetation Index) during the growing season of Aglianico grapevine. The data reported represent the mean and standard deviation of the values measured along the rows investigated during 15 UAV flights.

The analysis of vegetation indices (Clre, GNDVI, NDVI, and RENDVI) calculated from multispectral imagery acquired via UAV confirmed significant differences between CDI and RF treatments. Specifically, CDI treatment exhibited consistently higher mean values for all indices examined, as compared to RF (Figure 1). In particular, all indices (Clre, GNDVI, NDVI, and RENDVI) were significantly higher in irrigated than in rainfed plants, with values of 0.469 ± 0.094 vs. 0.409 ± 0.053 (*p* < 0.05), 0.689 ± 0.059 vs. 0.648 ± 0.037 (*p* < 0.05), 0.799 ± 0.062 vs. 0.749 ± 0.035 (*p* < 0.01), and 0.188 ± 0.032 vs. 0.168 ± 0.019 (*p* < 0.05), respectively.

**Figure 2:**
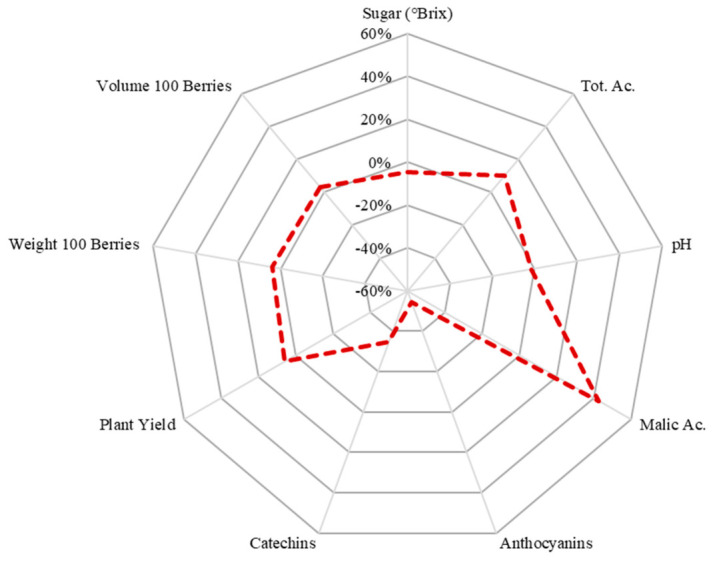
Spider plot showing the percentage differences in main grape characteristics at harvest between the irrigated and rainfed treatments.

Figure 2 describes the percentage differences in key grape characteristics at harvest between CDI and RF treatments. Malic acid exhibited the highest positive percentage difference (>42%), with a significant difference (*p* = 0.007) between irrigated (1.78 g/L) and rainfed (1.25 g/L) treatments. Conversely, anthocyanins and catechins measured through colorimetric assays showed significant negative percentage differences (−60% and −30%, respectively) with *p*-values of 0.002 and 0.004, respectively. The rainfed treatment contained 72.5 mg/L of anthocyanins and 52.0 mg/L of catechins, whereas the irrigated treatment had lower concentrations, being 33.2 and 34.0 mg/L, respectively.

Plant yield, along with related parameters such as the weight and volume of 100 berries showed no significant differences between treatments, with percentage variations close to 0%, although irrigated treatment always showed slightly higher values as compared to rainfed (3231.7 g ± 1338.6 vs. 3054.2 g ± 1136.6 for yield; 270.9 g ± 18.7 vs. 260.9 g ± 11.6 for weight of 100 berries; 245.0 cm^3^ ± 18.4 vs. 237.5 cm^3^ ± 10.4 for volume of 100 berries). Other quality factors such as pH, total acidity and sugar content (°Brix) exhibited very similar average values between the two treatments (8.06 g/L ± 0.32 in irrigated treatment and 7.32 g/L ± 0.87 in rainfed for total acidity; 3.16 ± 0.06 in irrigated treatment and 3.21 ± 0.06 in rainfed for pH; 24.7 °Brix ± 0.95 and 23.6 °Brix ± 1.02 for rainfed and irrigated treatment, respectively, in the case of sugar content).

### 2.2. Metabolomic Profile

**Figure 3:**
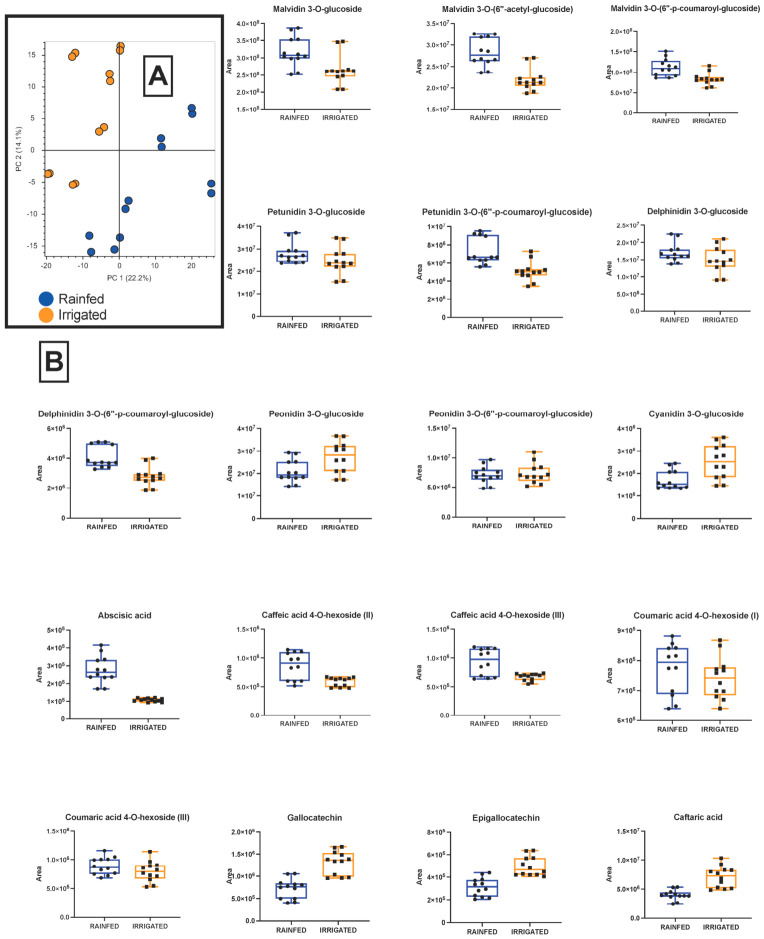
Untargeted metabolomic analysis of grapes from rainfed and irrigated Aglianico plants. Panel (**A**). Principal component analysis on Aglianico grapes obtained from the experimental trial reporting the sample grouping in both positive and negative ion mode, explaining an overall 36.3% of the total variance. Panel (**B**). Relative quantitative changes in specific metabolites, as measured in grape samples from rainfed (blue) and irrigated (orange) grapevines. Analytical performances along with ratio, log fold change and *p*-value upon discriminant analysis of the compounds are reported in Supplementary Table S2.

Metabolomics was already used to recognize phytochemical signatures associated with drought stress in grapevines [1,15]. Accordingly, metabolomics here revealed a significant differentiation in the secondary metabolite profile between Aglianico grape samples from rainfed and irrigated plants. Principal Component Analysis (PCA) showed a clear separation (clustering) of the samples based on the water regime, indicating an evident impact of irrigation on the grape phytometabolite representation (Figure 3A). Beyond the overall variance, the two conditions resulted in a unique molecular pattern, reflecting modifications of specific biosynthetic pathways. Through this analysis, several metabolic classes were identified, including anthocyanins, phenolic compounds, stilbenes, flavanols, flavanones, and other phytometabolites, as detailed in Supplementary Table S2.

In RF samples, a significant over-representation of anthocyanins was observed, particularly of malvidin 3-*O*-glucoside, petunidin-3-*O*-glucoside, delphinidin 3-*O*-glucoside, and their coumaroylated and acetylated forms, confirming the results from the colorimetric assays. In addition, other compounds showed an increased abundance in RF samples, including hydroxycinnamic acid derivatives, such as isomers of coumaroyl-hexose and caffeoyl-hexose. Finally, abscisic acid (ABA) was found at a higher concentration in RF grapes.

Conversely, CDI samples exhibited different metabolomic characteristics. Flavan-3-ols such as gallocatechin and epigallocatechin, precursors of condensed tannins, and isomers of gallic acid hexoside showed higher levels compared to RF samples. A similar quantitative trend was observed for the hydroxycinnamic acid derivatives caffeoyl-malic acid and caftaric acid, as well as for the isomers of glucosyl-trihydroxystilbene. Besides the polyphenol classes, the untargeted analysis indicated a higher statistically significant abundance of other phytometabolites in irrigated grapes, such as ascorbic acid and pipecolic acid (Supplementary Table S2, *p*-value < 0.05).

### 2.3. Telomere Length Analysis

qPCR amplification efficiencies were 97.3% for telomere and 98.6% for the single-copy gene (SCG). Cq values showed high reproducibility, with a standard deviation of less than 0.5. Two independent standard curves were generated for telomere and SCG sequences, with SYBR Green fluorescence signals acquired at 60 °C for telomeres and 64 °C for SCG during each qPCR cycle.

Differences in TL between RF and CDI treatments were analyzed using a linear mixed-effects model including treatment, time, and the treatment × time interaction as fixed effects, with plant ID included as a random intercept to account for repeated measurements within the same plant. The analysis included 93 available TL observations from 47 plants; 46 plants had complete paired measurements at both time points.

Observed mean TL values were 1.24 ± 0.31 at t0 and 0.87 ± 0.25 at t1 in CDI plants, and 1.12 ± 0.37 at t0 and 0.56 ± 0.30 at t1 in RF plants. In the mixed-effects model, TL did not differ significantly between treatments at t0 (RF vs. CDI: estimate = −0.117, *p* = 0.198), indicating comparable baseline TL values. TL decreased significantly from t0 to t1 in CDI plants (estimate = −0.370, *p* < 0.001) and in RF plants (estimate = −0.568, *p* < 0.001). At t1, RF plants showed significantly lower TL values than CDI plants (estimate = −0.314, *p* = 0.0009).

**Figure 4:**
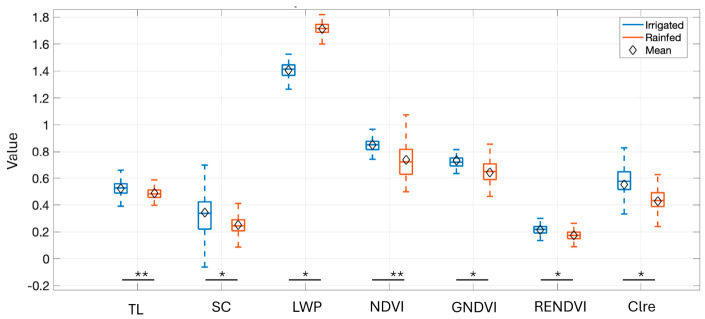
Boxplot comparison of key physiological and spectral parameters under controlled deficit irrigation (CDI) and rainfed (RF) treatments. Variables include Telomere Length (TL), Stomatal Conductance (SC), Leaf Water Potential (LWP), Chlorophyll Index Red Edge (ClRE), Green Normalized Difference Vegetation Index (GNDVI), Normalized Difference Vegetation Index (NDVI), and Red Edge Normalized Difference Vegetation Index (RENDVI). TL refers to measurements obtained at the final sampling time (t1). For TL, statistical inference was based on the planned between-treatment contrast derived from the linear mixed-effects model; for physiological and spectral parameters, between-treatment comparisons were assessed as described in the Statistical Analysis Section. LWP values refer to the ripening stage and are presented as absolute values for clarity, with higher values indicating greater water stress. Asterisks denote statistically significant differences between treatments (* *p* < 0.05; ** *p* < 0.01).

The treatment × time interaction suggested a greater TL decrease in RF plants compared with CDI plants, although this effect did not reach the conventional significance threshold in the primary model including all available observations (estimate = −0.197, *p* = 0.064). In a complete-pair sensitivity analysis, the treatment × time interaction was significant (estimate = −0.221, *p* = 0.036), supporting the pattern of a larger TL reduction in RF plants. These results indicate that TL variation was associated with irrigation regime over time and support a stronger telomere length reduction under rainfed conditions. Independent analyses by using physiological (LWP, gs), imaging (vegetation indices), and metabolic markers further reinforced this evidence, showing coherent patterns of stress response across multiple layers of independent plant measurements (Figure 4).

## 3. Discussion

Detecting and characterizing plant responses to drought stress is increasingly important for sustainable agriculture under climate change scenarios [1]. Grapevines are continuously exposed to fluctuations in water availability that can affect physiological activity, berry composition, and fruit quality. In the present study, physiological measurements, UAV-derived vegetation indices, berry metabolomic profiling, and TL analysis consistently differentiated grapevines subjected to rainfed and controlled deficit irrigation treatments under field conditions. Together, these observations support the occurrence of distinct plant responses associated with the two irrigation regimes.

Physiological analyses showed more negative leaf water potential and lower stomatal conductance in rainfed vines compared with irrigated plants, indicating a more pronounced water deficit condition during the growing season. These findings were consistent with UAV-derived vegetation indices, which reflected differences in canopy vigor and physiological status between treatments. Metabolomic profiling further supported the occurrence of different stress-associated responses in berries collected from rainfed and irrigated vines. In particular, rainfed samples showed increased accumulation of anthocyanins and abscisic acid (ABA), compounds commonly associated with drought responses and oxidative stress conditions in grapevine [2,5,6]. Anthocyanin accumulation is considered part of the protective response against oxidative and photo-oxidative damage, while ABA is a key phytohormone involved in drought signaling pathways and stomatal regulation. In addition to its central role in drought responses, ABA interacts with other hormonal pathways, including auxin-mediated signaling, contributing to the regulation of stomatal behavior and plant adaptation to water deficit conditions. Recent studies have highlighted the importance of auxin–ABA crosstalk in coordinating drought-responsive signaling networks and optimizing plant responses to environmental stress [16].

In contrast, berries from irrigated vines showed a different metabolomic profile characterized by higher levels of specific flavan-3-ols and gallic acid derivatives. These compounds may reflect different metabolic adjustments associated with water availability and fruit development under controlled irrigation conditions. Altogether, the metabolomic results were coherent with the physiological measurements and confirmed that the two irrigation regimes induced different plant responses during the experimental period.

More importantly, the mixed-effects analysis showed that TL varied over time in association with the irrigation regime. No significant difference between RF and CDI plants was detected at baseline, whereas RF plants showed significantly lower TL values than CDI plants at the end of the growing season. TL decreased significantly in both treatments, but the estimated reduction was larger in RF plants. The treatment × time interaction suggested a stronger TL decline under rainfed conditions, although this effect did not reach the conventional significance threshold in the primary model including all available observations. However, the complete-pair sensitivity analysis supported this pattern, showing a significant treatment × time interaction. These findings strengthen the evidence that TL variation was associated with prolonged exposure to contrasting irrigation conditions under field settings, while also indicating that the interaction effect should be interpreted cautiously (Supplementary Table S3).

Previous investigations in plants suggested that telomere dynamics may be influenced by environmental and oxidative stress conditions. Drought stress is known to activate complex ROS-mediated signaling networks that regulate physiological, metabolic, and molecular responses in plants. Reactive oxygen species function not only as potentially damaging molecules but also as key regulators of stress perception, stomatal behavior, hormonal signaling, and redox homeostasis. Recent evidence highlights the central role of ROS signaling in coordinating plant adaptation to water deficit and integrating stress responses across different cellular compartments [17]. In this context, the lower TL values observed in rainfed vines may be associated with a physiological environment characterized by enhanced oxidative pressure, although direct measurements of ROS accumulation or oxidative DNA damage were beyond the scope of the present study.

Studies conducted in Arabidopsis thaliana reported associations between oxidative stress and telomere shortening [8], while investigations in almond and poplar highlighted possible relationships between telomere dynamics, aging, and environmental responses in perennial species [9,10]. In addition, natural variation in telomere length has been associated with adaptive and developmental traits in several plant species [11]. The present study extends these observations to field-grown grapevines exposed to contrasting irrigation regimes. However, the mechanisms linking environmental stress and TL variation in plants remain incompletely understood.

The TL differences observed in this study were consistent with the physiological and metabolomic responses detected between rainfed and irrigated vines. Nevertheless, the present results should be interpreted cautiously. Oxidative stress markers, telomerase activity, and DNA damage parameters were not directly measured, preventing mechanistic interpretation of telomere shortening under drought conditions. Consequently, the observed TL variation should be considered as an association with irrigation treatments rather than direct evidence of specific molecular mechanisms. In addition, the study was conducted on a single grapevine cultivar under a specific environmental context, and further investigations are required to evaluate whether similar TL responses occur across different cultivars, vineyards, and climatic conditions.

Quantitative PCR-based TL analysis represents a rapid and relatively inexpensive molecular approach that can be integrated with physiological and metabolomic measurements in field studies. In the present work, the qPCR-based protocol successfully detected TL differences between irrigation treatments and showed consistency with established indicators of plant water status. Although additional validation is required, integrating molecular approaches with physiological and remote sensing analyses may contribute to a more comprehensive characterization of grapevine responses to water availability under field conditions.

Overall, this study provides preliminary evidence of an association between irrigation regime and TL variation in field-grown grapevine. The integration of physiological measurements, UAV-derived vegetation indices, metabolomic profiling, and TL analysis highlighted coherent differences between rainfed and irrigated vines during the growing season. Future studies should investigate the molecular mechanisms potentially underlying TL variation under drought conditions by integrating telomere measurements with direct assessments of reactive oxygen species (ROS), telomerase activity (e.g., TERT expression), and DNA damage markers. In addition, repeated drought events across consecutive growing seasons should be evaluated to determine whether they result in cumulative TL reduction and whether such changes are associated with alterations in vine performance, berry composition, fruit quality traits, or long-term vineyard productivity. Such multi-year longitudinal studies could help clarify both the mechanistic basis and the biological significance of telomere dynamics in perennial crops subjected to recurrent environmental stress.

## 4. Materials and Methods

### 4.1. Experimental Design

This study was conducted in a 2-hectare Aglianico vineyard belonging to the Tenuta Donna Elvira winery in Montemiletto (AV, Italy), where a long-term experiment is being carried out on the effects of controlled deficit irrigation on the response of Aglianico vines. In the vineyard, viticultural zoning was realized based on the spatial variability of soil assessed by a pedological survey supported by geophysical measurements, and two homogeneous zones were defined (Hz1 and Hz2).

**Figure 5:**
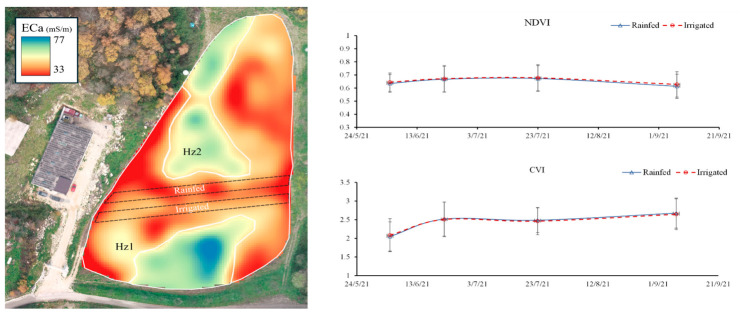
Experimental vineyard layout, soil spatial variability, and study area selection. The left panel shows the soil apparent electrical conductivity (ECa, mS/m) map derived from a geophysical survey, which, in accordance with the pedological survey, allowed the delineation of two main homogeneous soil zones (Hz1 and Hz2). The Hz1 zone was selected for the experiment. To overcome potential issues related to soil micro-variability within this macro-zone, historical UAV-derived vegetation indices (NDVI and Chlorophyll Vegetation Index—CVI, right panels) acquired during the 2021 growing season were used as a spatial screening tool. By analyzing these data, specific adjacent rows exhibiting highly uniform canopy vigor were identified, demonstrating a negligible effect of local soil micro-variability on vine performance. The experimental rainfed (RF) and controlled deficit irrigation (CDI) plots were subsequently established precisely along these selected rows.

To minimize the influence of soil variability on plant responses, the experiment was conducted within the Hz1 vineyard zone. Two experimental plots (RF and CDI) were established within this area. To further reduce the impact of spatial soil micro-variability on plant performance, a 2021 UAV survey was used to select the most homogeneous vineyard rows (four rows per treatment) where differences in plant response were considered negligible (Figure 5).

The Soil–Plant-Atmosphere (SPA) system was continuously monitored throughout the experimental period using a DAVIS weather station installed in the center of the vineyard to record environmental parameters, including temperature, rainfall, wind speed, and solar radiation. The reference evapotranspiration was calculated according to Penman-Monteith [18,19]. In addition, three monitoring nodes equipped with TDR probes were installed in each experimental plot to measure soil water content at different depths (15, 35, and 65 cm). A remotely managed drip irrigation system equipped with compensated pipes was installed in the CDI treatment. Irrigation management aimed to maintain midday LWP within a range between −1.2 and −1.5 MPa. Irrigation timing and frequency were determined through direct LWP measurements, whereas irrigation volumes were calculated based on SPA system data to compensate for atmospheric evapotranspiration demand.

Physiological measurements, UAV-based vegetation indices, berry metabolomic analyses, and TL determinations were performed during the growing season under field conditions. Sampling was conducted at two time points: t0 (6 June, before flowering) and t1 (29 August, before harvest). Each grapevine was considered an independent biological replicate for physiological and molecular analyses, while qPCR measurements were performed in technical triplicate for each biological sample.

### 4.2. Plant Monitoring

During the vine growth period (May to September), physiological measurements in terms of midday LWP and stomatal conductance were carried out ten times.

LWP was measured by using a Scholander pressure bomb (SAPS II, 3115, Soilmoisture Equipment Corp., Santa Barbara, CA, USA). The measurements were taken on six vines by selecting well-expanded and sun-exposed leaves (1 leaf/vine) for each treatment, and started at 12:00 until 13:00 (solar time). Stomatal conductance (gs) was acquired by a LI-600 porometer (LI-COR model 600, LI-COR Biosciences, Lincoln, NE, USA). The measurements were taken on six vines by selecting well-expanded and sun-exposed leaves (3 leaves/vine) for each treatment, and started at 11:00 until 12:00 (solar time).

Furthermore, the plant response to water supply was assessed by using a UAV multispectral flight (DJI Phantom multispectral, five bands: blue, green, red, red edge, and near-infrared-NIR) conducted during the growing season from May to September 2024 (15 flights). The images collected were processed with PIX4D Mapper software (4.10 version) to calculate the following four vegetative indices: Clre, GNDVI, NDVI, and RENDVI [20].

The Chlorophyll Index Red Edge (Clre) is a vegetation index that estimates chlorophyll content in leaves by using the ratio of reflectivity in the near-infrared (NIR) and red-edge bands. High values of the Clre indicate a significant concentration of chlorophyll in the plants, which is typically associated with good health and vigorous vegetative growth. Conversely, low Clre values may suggest potential challenges, such as nutrient deficiencies, water stress, or susceptibility to pathogens. It is calculated as follows:

Clre = (NIR/Red Edge) − 1(1)

The Green Normalized Difference Vegetation Index (GNDVI) is an index that gives a measure of plant “greenness” or the photosynthetic activity, as it is very sensitive to chlorophyll concentration. It utilizes the near-infrared (NIR) and green bands of the electromagnetic spectrum. The index ranges from −1 to 1, with higher values indicating denser and healthier vegetation, and it is calculated as follows:

GNDVI = (NIR − Green)/(NIR + Green)(2)

The Normalized Difference Vegetation Index (NDVI) measures the amount of live green vegetation in an area, calculated from the visible and near-infrared light reflected by vegetation. The index ranges from −1 to 1, with higher values indicating denser and healthier vegetation, and it is calculated as follows:

NDVI = (NIR − red)/(NIR + red)(3)

The Red Edge Normalized Difference Vegetation Index (RENDVI) is similar to NDVI but uses the ratio of near-infrared and the edge of red light. It is particularly effective for detecting changes in biomass, chlorophyll content, and vegetation stress, offering advantages over traditional, red-based indices in dense vegetation conditions. The index ranges from −1 to 1, with higher values indicating healthier vegetation, and it is calculated as follows:

RENDVI = (NIR − Red Edge)/(NIR + Red Edge)(4)

### 4.3. Vine Yield and Physicochemical Characterization of Berries

Under both RF and CDI treatments, six representative vines per plot were harvested on 27 September 2024. Total yield per vine was recorded, and a randomly selected sub-sample of 100 berries per vine was used to determine average berry weight and volume. From the harvested vines, representative berry samples were collected and divided into aliquots. A portion was immediately separated and stored for subsequent metabolomic and molecular profiling. The remaining berries were used to determine sugar (Brix°), pH, total acidity, and the concentrations of anthocyanins, catechins, and malic acid. These analyses were performed using a Flash automatic titrator and a Hyperlab Smart automated analyzer (Diacron International, Grosseto, Italy), a system that executes enzymatic and colorimetric assays with automated sample and reagent handling in disposable cuvettes [21,22,23].

### 4.4. Untargeted Metabolomics of Berries

A comparative untargeted metabolomic analysis of the grape samples from vines undergoing RF and CDI treatments was performed using high-resolution liquid chromatography-tandem mass spectrometry (LC-HR-MS/MS). All reagents and standard compounds were mass spectrometry grade (Merck, Darmstadt, Germany). Grape samples (6 biological replicates for each treatment) were freeze-dried and pulverized. Metabolites were extracted from 100 mg of powder with 3 mL of a methanol/water/formic acid solution (70/29.9/0.1, *v*/*v*/*v*), following a protocol [22] adapted to grape samples. The extraction involved ultrasonication (15 min, 20 °C), vortexing (5 min, 750 rpm), and centrifugation (18,000× *g*, 5 min, 4 °C). The resulting supernatants were diluted 1:5 in water. LC-HR-MS/MS analyses were performed in technical duplicate using a Vanquish LC system coupled to an Orbitrap Exploris 120 mass spectrometer (Thermo Fisher Scientific, Bremen, Germany). Chromatographic separation was achieved on a Kinetex biphenyl column (100 × 2.1 mm, 2.6 µm; Phenomenex, Torrance, CA, USA) at 35 °C. The mobile phase consisted of 0.1% formic acid in water (solvent A) and acetonitrile (solvent B) at a flow rate of 0.3 mL/min. The elution gradient was as follows: 5% B for 0.5 min, increased to 15% at 1.5 min, 40% at 10 min, 70% at 15 min, and 95% from 16 to 19 min. The mass spectrometer operated in positive/negative polarity switching mode over the m/z range of 120–1500 with a resolution of 60,000 (FWHM at *m*/*z* 200). Key heated ESI source parameters were: spray voltage ±3.5/−3.2 kV; ion transfer tube temperature 300 °C; vaporizer temperature 320 °C. Mass accuracy was maintained using internal calibration (EASY-IC) before each run. Compound identification was achieved using a polarity-switching acquisition mode and by matching mass and fragmentation spectra against in-house spectral databases. Quality control (QC) samples were prepared by pooling aliquots from all samples, and a procedural blank was processed in parallel for background correction. Raw metabolomics data were processed with Compound Discoverer 3.3 (Thermo Fisher Scientific) using an untargeted metabolomics workflow that included retention time alignment, background subtraction, and feature detection. Identification was performed by analyzing QC samples in data-dependent MS2 (ddMS2) mode with stepped normalized collision energies of 20, 45, and 60%. Features (exact mass, formula, and fragmentation spectra) were matched against spectral libraries (mzCloud, mzVault) and online databases (ChemSpider, KEGG, and Phenol-Explorer). Differential analysis was conducted using Principal Component Analysis (PCA) and Volcano Plots, with *p*-values adjusted using the Benjamini–Hochberg algorithm.

### 4.5. qPCR

Genomic DNA was isolated from grapevine leaf samples using the Dneasy Plant Mini Kit (QIAGEN, Hilden, Germany). The DNA concentration and purity were assessed for each sample using a NanoDrop ND-1000 spectrophotometer (Thermo Scientific, Wilmington, DE, USA). Before qPCR analysis, all the genomic DNA samples were diluted to a final concentration of 10 ng/μL. DNA quality requirements included a concentration yield > 30 ng μL, a 260/280 ratio > 1.7, and a 260/230 ratio > 1.8. All qPCR analyses were performed according to established guidelines for qPCR experiments and telomere length measurements [12,13,24]. TL was measured using a CFX96 Real-Time PCR Detection System (Bio-Rad, Hercules, CA, USA) in a total reaction volume of 20 μL containing 30 ng of genomic DNA, iTaq Universal SYBR Green Supermix (Bio-Rad, Hercules, CA, USA), and forward and reverse primers targeting telomeric repeats and the SAND.1 SCG. Telomere primers were selected according to [25], whereas SAND.1 primers were derived from [26]. Primer sequences and amplicon characteristics are reported in Supplementary Table S1.

Several candidate SCG primer pairs were initially evaluated to identify the most suitable reference gene based on amplification specificity and efficiency. Among the tested primer pairs, SAND.1 was selected for subsequent TL analyses. Several candidate loci were initially screened (Supplementary Table S1), and SAND.1 was selected based on its amplification specificity, expected amplicon size, and overall qPCR performance. Specific amplification was confirmed by agarose gel electrophoresis and melting curve analyses (Supplementary Figure S1). Amplification specificity was verified by melting curve analysis and agarose gel electrophoresis of PCR products (Supplementary Figure S1). The telomere amplicon showed the expected molecular size (~71 bp), while the SAND.1 amplification product corresponded to the expected size (~161 bp), confirming assay specificity.

Each biological sample was analyzed in technical triplicate within two independent qPCR runs, including no-template controls (NTCs). For each biological sample, mean cycle threshold (Ct) values from technical triplicates were used to calculate relative telomere length (TL). Standard curves were generated for each primer pair to evaluate amplification efficiency and linearity. The thermal cycling conditions consisted of an initial denaturation step at 95 °C for 3 min, followed by 2 cycles at 95 °C for 30 s and 32 amplification cycles, including 95 °C for 30 s, 60 °C for 30 s with signal acquisition, and 64 °C for 30 s with signal acquisition. Melting curves were generated at the end of amplification by increasing the temperature from 60 to 95 °C in 0.5 °C increments.

Relative telomere length was expressed as the telomere-to-single-copy gene ratio (T/S ratio) and calculated using the ΔΔCt method with Pfaffl correction [27].

### 4.6. Statistical Analysis

Physiological, metabolomic, and TL data obtained from RF and CDI treatments were statistically analyzed to evaluate differences between irrigation regimes during the experimental period. Data are presented as mean ± standard deviation (SD).

TL data were analyzed using a linear mixed-effects model to account for the repeated-measurements structure of the experiment. TL was included as the dependent variable, while treatment, time, and the treatment × time interaction were included as fixed effects. Plant ID was included as a random intercept to account for within-plant correlation between t0 and t1 measurements. Treatment and time were modeled as categorical variables, with CDI and t0 used as reference categories. The treatment × time interaction was used to formally test whether TL changes over time differed between RF and CDI plants. Model-based planned contrasts were used to compare treatments at each time point and to estimate within-treatment changes over time. The model was fitted using restricted maximum likelihood, and statistical inference for fixed effects was based on Satterthwaite degrees of freedom. A complete-pair sensitivity analysis was also performed by refitting the same model in plants with available TL measurements at both t0 and t1. Statistical significance was set at *p* < 0.05 (See Supplementary Table S3). For qPCR analyses, each biological sample was analyzed in technical triplicate, and mean Ct values from technical replicates were used for TL calculation before statistical modeling. Each grapevine was considered an independent biological replicate for physiological and molecular analyses.

Vegetation indices derived from UAV multispectral imagery and metabolomic datasets were integrated with physiological and TL measurements to characterize grapevine responses under contrasting water regimes. Statistical analyses and graphical representations were performed using standard statistical software.

## 5. Conclusions

RF vines showed distinct physiological and metabolic responses compared with CDI plants, including more negative leaf water potential, lower stomatal conductance, and increased accumulation of stress-associated metabolites such as anthocyanins and abscisic acid.

TL decreased over the growing season and was lower in RF vines than in CDI vines at the final sampling time. These findings, supported by physiological, spectral, and metabolomic responses, indicate an association between plant water stress and telomere dynamics under field conditions and highlight the potential of TL as a genomic indicator of grapevine wellness. The present work represents a preliminary field-based investigation of TL variation in grapevine under contrasting irrigation conditions. However, additional studies including different cultivars, environmental conditions, and mechanistic analyses will be necessary to better clarify the biological significance of telomere dynamics and their relationship with plant stress responses.

## Data Availability

The original contributions presented in this study are included in the article/Supplementary Materials. Further inquiries can be directed to the corresponding author.

## Supplementary Materials

The following supporting information can be downloaded at: https://www.mdpi.com/article/10.3390/plants15131988/s1.
