# Supplementary material for: Association Between Contrasting Water Regimes and Telomere Length Variation in Field-Grown Grapevines: An Integrated Physiological, Metabolomic and Molecular Approach
Source: Plants (Basel). 2026 Jun 26;15(13):1988. doi: 10.3390/plants15131988 (PMC13363928; doi:10.3390/plants15131988)
Supplement: Supplementary file 1 [file plants-15-01988-s001.zip › plants-4355457-supplementary.pdf]

Supplementary Table S1. Grapevine gene primer sequences used to identify the most suitable expressed genes in this study. All primer pairs reported in the table were initially tested. The primer pairs shown in bold were selected and used for the final expression analysis based on their amplification efficiency and specificity. For telomere analysis, a single primer pair was used for all samples.

Gene Symbol	Forward primer sequence (*5′*to *3′)*	Reverseprimer sequence (*5′*to *3′)*	Amplicon (bp)	GENE ID	Source
GAPDH	TGGAGACAAGAAACAGCACCCT	CGGCAATTCCGCCATTTAAC	112	100233024	Yu, 2019
BES-1	TCAGCAAGTCCATGCTCATC	TGGCATCAGCACTAGCATTC	178	100263555	Wang, 2023
TIP2;1	GGAGGAAGAGCAAGTTGTGC	CACAGCTTGAACCAAAGCAA	115	100233028	Lukšic, 2023
SAND	AGCCAACTTGGTGGTTATGC	GGTGGTTATGCAAGGCAGTT	60	100265538	Reid, 2006
**SAND.1**	CCCTTTCCCCAAACTCTCTC	GGTGGTTATGCAAGGCAGTT	161	100265538	Reid, 2006
ACT	AACCCACACTGATCGTAGGC	GGACGGTCTCTGCTCTTCAC	162	100264704	Tashiro, 2016
**Telomere**	CCCCGGTTTTGGGTTTTGGGTTTTGGGTTTTGGGT	GGGGCCCTAATCCCTAATCCCTAATCCCTAATCCCT	71	100854718	Vaquero-Sedas 2014

Supplementary Figure S1. Agarose gel electrophoresis showing amplification products obtained using telomere-specific and SCG (SAND.1) primers on *Vitis vinifera* (cv. Aglianico) DNA. The lane on the left contains a 100 bp DNA ladder, with visible reference bands at 100 and 200 bp. The central lane shows the telomere amplification product (~71 bp), visible as a distinct band slightly below the 100 bp marker, with minor smearing attributed to expected primer-dimer formation or secondary structures common in telomeric sequences. The right lane shows the single-copy gene (SCG) SAND.1 amplicon (~161 bp), confirming the specificity and performance of the qPCR assay.

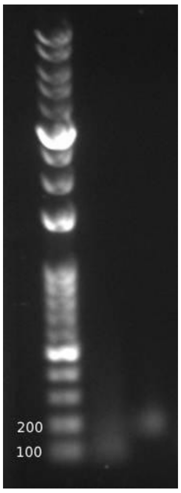

Supplementary Table S2. Annotated and identified compounds resulting from the metabolomic workflow in both positive and negative ion modes. The data summarize the annotated delta mass (ppm), calculated molecular weight (MW), mass-to-charge ratio (m/z), maximum area counts across all samples, and the ratio between irrigated and rainfed samples, along with the corresponding $\log_2$ fold change.

Name	Formula	DeltaMass	Calc. MW	m/z	RT [min]	Area (Max.)	Reference Ion	(irrigated) /	Fold Change:	(irrigated) / (rainfed)
acid	O6	-1,88	176,0318	177,039	0,764	561308,364	[M+H]+1	1,645	0,72	4,1679E-05
c acid	N O2	-0,72	129,0789	130,0862	0,808	86009446,9	[M+H]+1	1,525	0,61	0,00213373
acid	H16	0,54	332,0745	331,0673	1,022	3944149,97	[M-H]-1	1,379	0,46	0,00688804
acid	H16	0,65	332,0746	331,0673	1,171	1158207,62	[M-H]-1	1,328	0,41	0,07198427
acid	H16	0,59	332,0745	331,0673	1,282	2469651,83	[M-H]-1	1,778	0,83	9,4521E-10
acid	H16	0,33	332,0745	331,0672	1,442	12421140,7	[M-H]-1	1,306	0,39	0,00151155
echin	H14 O7	0,53	306,0741	305,0668	2,251	1664753,09	[M-H]-1	1,782	0,83	2,9747E-05
acid	H12 O9	0,56	312,0483	311,041	2,588	10330203	[M-H]-1	1,841	0,88	1,0355E-05
han	H12 N2	-0,55	204,0898	205,0971	2,687	7933968,19	[M+H]+1	0,645	-0,63	0,00443711
acid 4-O-	H18 O9	-0,69	342,0949	341,0876	2,829	828875,509	[M-H]-1	4,364	2,13	8,3117E-11
din 3-O-	H21	-1,63	465,1033	465,1025	2,897	22420646	[M]+	0,903	-0,15	0,10345879
acid 4-O-	H18 O9	-0,64	342,0949	341,0876	2,929	1143220,05	[M-H]-1	0,703	-0,51	0,0014041
acid 4-O-	H18 O9	-0,59	342,0949	341,0876	3,113	1190184,21	[M-H]-1	0,703	-0,51	0,00168861
catechin	H14 O7	0,47	306,0741	305,0668	3,114	638311,973	[M-H]-1	1,458	0,54	5,0399E-05
hexosid	H22	-0,07	466,1111	465,1039	3,19	604929,21	[M-H]-1	1,283	0,36	0,00760841
3-O-	H21	0,09	449,1084	449,1084	3,239	3615267,8	[M]+	1,626	0,7	0,00476406
hexosid	H22	-0,39	466,1109	465,1037	3,242	1539295,69	[M-H]-1	1,492	0,58	0,00101653
malic	H12 O8	-0,06	296,0532	295,0459	3,254	3170358,37	[M-H]-1	1,9	0,93	6,4394E-05
c acid 4-	H18 O8	-0,05	326,1002	325,0929	3,26	881233,763	[M-H]-1	0,941	-0,09	0,50585397
n 3-O-	H23	-1,69	479,119	479,1181	3,27	37121379,6	[M]+	0,889	-0,17	0,11599821
c acid 4-	H18 O8	0,36	326,1003	325,093	3,327	1401056,07	[M-H]-1	8,1	3,02	3,1672E-08
c acid 4-	H18 O8	-0,24	326,1001	325,0928	3,397	1158362,24	[M-H]-1	0,93	-0,1	0,08705747
n 3-O-	H23	-1,66	463,124	463,1233	3,615	36765010,6	[M]+	1,453	0,54	0,02112515
3-O-	H25	-2,23	493,1346	493,1335	3,626	386806896	[M]+	0,852	-0,23	0,00559259
oxyflava none-C-	H22 O12	0,19	466,1112	465,1039	4,313	567008,182	[M-H]-1	2,903	1,54	0,00015866
3-O-(6-O-	H27	-1,44	535,1452	535,1444	4,796	32579759,5	[M]+	0,768	-0,38	1,4954E-05
trihydrox	H22 O8	-0,26	390,1314	435,1296	4,995	2409670,05	H]-1	1,806	0,85	3,3079E-05
din-3-O- (6-p-	H27 O14	-1,44	611,1401	611,1392	5,01	5112842,21	[M]+	0,734	-0,45	0,00013113
n 3-O-(6- O-p-	H29 O14	-1,62	625,1557	625,1547	5,592	9521629,16	[M]+	0,77	-0,38	0,00034244
trihydrox	H22 O8	-0,28	390,1314	389,1241	5,855	1642826,41	[M-H]-1	2,855	1,51	5,3989E-05
3-(6-O-p-	H31	-2,07	639,1714	639,1701	6,209	151078795	[M]+	0,768	-0,38	0,00142282
n 3-(6-	H29	-1,69	609,1608	609,1598	6,22	10996749,1	[M]+	1,002	0	0,75553487
acid	H20 O4	0,18	264,1362	263,1289	6,708	415824,353	[M-H]-1	0,407	-1,3	7,1627E-08

Supplementary Table 3. Estimates refer to RF relative to CDI and to t1 relative to t0. Negative estimates indicate lower TL values or greater TL reduction in RF plants. SE indicates the standard error of the model-based estimate or contrast, and was used to derive the corresponding test statistic, confidence interval, and p-value. The primary model included all available observations and plant ID as a random intercept. The complete-pair sensitivity analysis included only plants with both t0 and t1 TL measurements.

Contrast	Estimate	SE	95% CI	*p*-value
RF vs CDI at t0	−0.117	0.090	−0.296 to 0.062	0.198
CDI, t1 vs t0	−0.370	0.070	−0.512 to −0.229	<0.001
RF, t1 vs t0	−0.568	0.076	−0.721 to −0.414	<0.001
RF vs CDI at t1	−0.314	0.091	−0.495 to −0.133	0.0009
Treatment × time interaction	−0.197	0.104	−0.406 to 0.012	0.064
Treatment × time interaction, complete-pair sensitivity	−0.221	0.102	−0.423 to −0.018	0.036
